# Immune-tolerance to human iPS-derived neural progenitors xenografted into the immature cerebellum is overridden by species-specific differences in differentiation timing

**DOI:** 10.1038/s41598-020-79502-9

**Published:** 2021-01-12

**Authors:** Giulia Nato, Alessandro Corti, Elena Parmigiani, Elena Jachetti, Daniele Lecis, Mario Paolo Colombo, Domenico Delia, Annalisa Buffo, Lorenzo Magrassi

**Affiliations:** 1 Department of Neuroscience Rita Levi-Montalcini, University of Turin, Via Cherasco 15, Torino, Italy; 2 Neuroscience Institute Cavalieri Ottolenghi (NICO), 10043 Orbassano, Torino Italy; 3 Department of Research, Fondazione IRCCS Istituto Nazionale Tumori, Milano, Via Amadeo 42, 20133 Milano, Italy; 4 IFOM, FIRC Institute of Molecular Oncology, Via Adamello 16, 20139 Milano, Italy; 5 Neurosurgery, Department of Clinical, Surgical, Diagnostic and Pediatric Science, University of Pavia, Foundation IRCCS Policlinico San Matteo, Pavia, Italy; 6 Istituto Di Genetica Molecolare IGM-CNR, via Abbiategrasso 207, 27100 Pavia, Italy

**Keywords:** Neuroscience, Development of the nervous system, Regeneration and repair in the nervous system

## Abstract

We xeno-transplanted human neural precursor cells derived from induced pluripotent stem cells into the cerebellum and brainstem of mice and rats during prenatal development or the first postnatal week. The transplants survived and started to differentiate up to 1 month after birth when they were rejected by both species. Extended survival and differentiation of the same cells were obtained only when they were transplanted in NOD-SCID mice. Transplants of human neural precursor cells mixed with the same cells after partial in vitro differentiation or with a cellular extract obtained from adult rat cerebellum increased survival of the xeno-graft beyond one month. These findings are consistent with the hypothesis that the slower pace of differentiation of human neural precursors compared to that of rodents restricts induction of immune-tolerance to human antigens expressed before completion of maturation of the immune system. With further maturation the transplanted neural precursors expressed more mature antigens before the graft were rejected. Supplementation of the immature cells suspensions with more mature antigens may help to induce immune-tolerance for those antigens expressed only later by the engrafted cells.

## Introduction

A rapidly growing field of research is the in vivo modeling of neurogenetic and neurodegenerative diseases by transplantation into the central nervous system of experimental animals of neural precursors derived from human induced pluripotent stem cells (hiPSdNP) obtained from peripheral tissues of patients with specific neurologic conditions^1^. Xenotransplantation of human neural precursors derived from embryonic stem cells (hESCdNP) and hiPSdNP is essential in testing neural transplantation strategies at a preclinical stage^2^. Despite some successes the widespread use of xenotransplantation for modeling and treating neurological diseases is limited in several ways. One limitation is the immune-reaction induced in the immunocompetent host by the xenotransplanted cells and to the commitment of transplanted human neural precursors to the human developmental plan that is little influenced by the host microenvironment^3–5^. Survival of hNP has been achieved in immunocompetent animals by chronic immunosuppressive therapy with CyclosporineA^6^ or tacrolimus^7^. Immune rejection of human neural precursors (hNP) after orthotopic transplantation into animal models has been addressed by transplanting hNP into immunodepressed hosts^8,9^ or immunocompetent animals made immuno-tolerant to hiPSdNP^10^. However, both strategies have limitations when graft survival must exceed few months. For example, CyclosporinA requires daily i.p. injections and after 12–20 weeks it damages the health of the host animal and must be terminated^11^. Immunodeficient mice and rats also need special housing and their average survival after transplantation is significantly shorter than that of immunocompetent animals due to infections and neoplasms^12^. An alternative for successful xenotransplantation of hNP is inducing immuno-tolerance in the host to the donor cells by transplantation of the same cells before complete maturation of the host immune system^10^. According to classical immunological studies, active tolerance of xenotransplanted cells can be induced by exposing the host to cells of the same species of the donor before the complete development of the immune system^13^. In practice at least in mice and rats, the animal must be exposed to the cell of the graft before or immediately after birth^13^. When applied to hNP derived from the dissociation of fetal tissue or from the in vitro differentiation of embryonic stem cells (ESC), or human induced pluripotent stem cells (hiPS), immunotolerance was obtained after a single intraperitoneal injection of the hNP between postnatal day 0 and 5^10,14^. Survival of the cells injected for tolerance induction was limited to few days^14^ and the intracerebral graft was usually performed after 8 weeks^10^. Unfortunately, long term survival of the xenografted neural precursors into the adult brain after tolerance induction at birth, is not always warranted^15,16^. Wide variations in survival despite immunological attack of the graft have been described according to the species and the strain of the host^14^, the origin of the cells^15,17^ and even the region in the brain targeted by the xenograft^18^. According to Billingham the best results with immune-tolerance induction were obtained when the tolerance inducing cells were directly injected into the fetus^13^. Xenotransplantation of mouse NP into the developing rat brain gave rise to integrated cells that differentiated into neurons and glial cells demonstrating life long survival without immunosuppression^19^. hESC derived NP xenografted into the ventricle of E17–E18 SD rat fetuses, integrated into the host brain and showed signs of glial and neuronal differentiation^20^. Donor derived neurons were still present two months after the graft^20^. However, these results were obtained before the demonstration that cell fusion after xenotransplantation can occur in vivo^21–23^ and thus this possibility was not tested by the authors^20^. Long term survival of neurons and glial cells derived in vivo from undifferentiated hESC (Cyth 25) was also obtained after transplantation into the developing brain of E14 ICR (CD1) mouse fetuses^24^. In the same animals part of the cells did not integrate into the brain but formed clusters inside the ventricles in a less differentiated state^24^. The less differentiated cells inside clusters may have given rise to NP migrating and differentiating into new neurons and glial cells much later than the time of the graft. Transplantation of NP derived from hESC into the developing brain of E14.5 mouse fetuses confirmed that the engrafted cells may integrate and start their differentiation into neurons and glial cells, but in the absence of intraventricular clusters of less differentiated cells, the engrafted cells were not demonstrated after P28 in immunocompetent mice^25^. Finally, despite demonstration of long term survival and differentiation of hiPSdNP in immunodepressed animals, no data are available on long term survival and differentiation of the same cells xenografted into the developing brain before maturation of the host immune-system. Altogether the above results show that it is unclear if hNP xenotransplanted into the developing brain of mouse and rats before the final differentiation of the host immune system could induce a level of immunotolerance that is going to allow the long-term survival of their descendants in the host.

We therefore asked if hiPSdNP can integrate, differentiate and persist indefinitely without rejection after xeno-transplantation in utero before the complete maturation of the host immune system. We found that hiPSdNP after in utero engraftment in mice and rats survived up to the first month of postnatal life of the host. Thereafter, we no longer found transplanted cells, suggesting that the xenografted hiPSdNP were rejected after an initial period when they migrated from the transplant, integrated into the tissue and started to differentiate in the host CNS. In contrast, the same cells survived indefinitely in the host CNS when transplanted into NOD-SCID mice. We propose that in utero engrafted hiPSdNP, contrary to what observed when xeno-transplanting NP derived from other rodent species, induced only incomplete tolerance despite being transplanted before the complete differentiation of the host immune system because they retained their normal differentiation timing thus starting the display of more differentiated antigens only when the host immune system was already mature. In agreement with this interpretation we also show that when we co-grafted in utero in the developing mouse cerebellum hiPSdNP together with a cell and protein suspension obtained from the adult cerebellum the long term survival of hiPSdNP was improved.

## Results

### Survival of hiPSdNP grafted in utero does not differ between rats and mice

**Figure 1:**
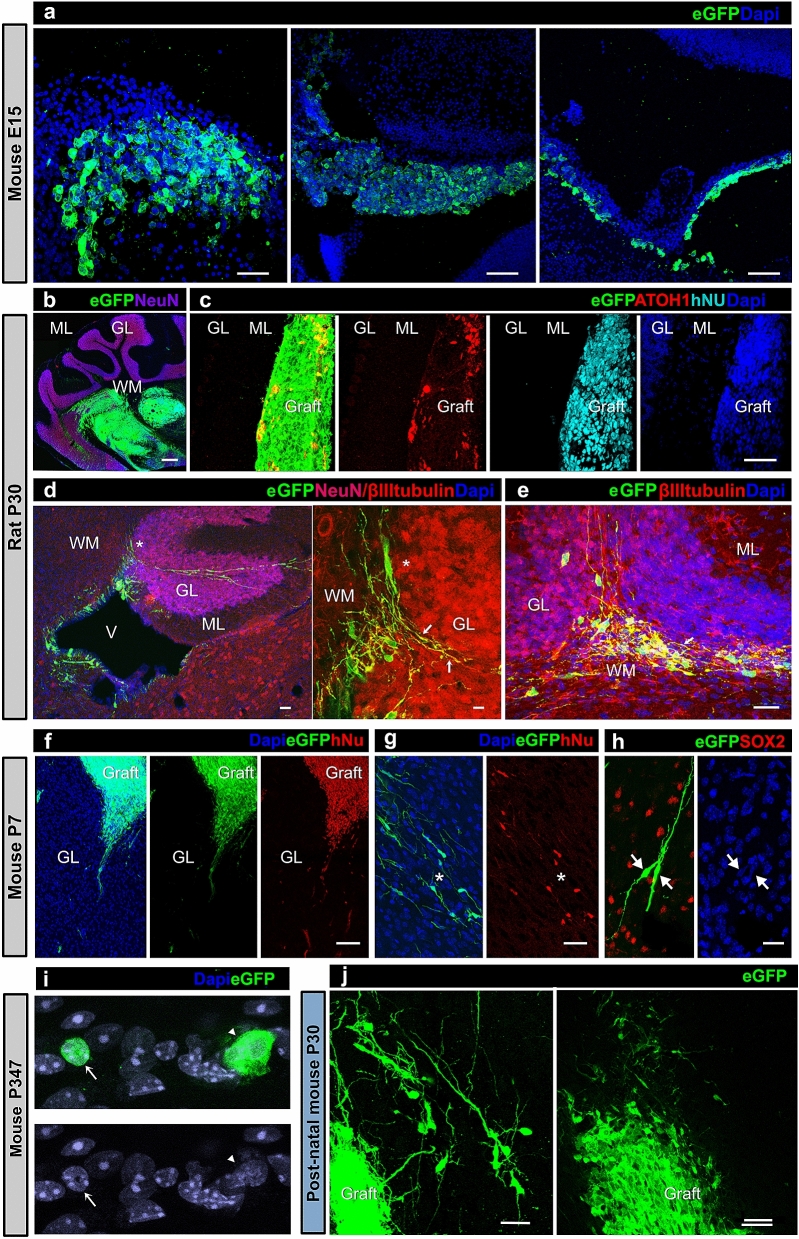
Representative photomicrographs of hiPSdNP transplanted in utero into the developing cerebellum of CD-1 mice (**a**, **f**, **g**, **h** and **j**) or Wistar rats (**b**, **c**, **d**, and **e**). Results of hiPSdNP transplantation in neonatal CD1 are shown in (*i*). All receiving animals were fully immunocompetent. (**a**) Embryos were collected at E15 the day after the transplant in utero. Three examples of hiPSdNP expressing eGFP engrafted in sagittal sections of the developing cerebellar and brainstem primordium: in the first image hiPSdNP are disperse in the developing cerebellar cortex (scale bar: 15 µm), in the second the engrafted cells are associated to the developing choroid plexus of the IV ventricle. (scale bar: 40 µm), the engrafted cells are located below the ependyma of the fourth ventricle (scale bar: 30 µm). (**b**) Rat cerebellum P30, the in utero grafted hiPSdNP cells are mostly located in the white matter core of the cerebellum close to the deep cerebellar nuclei. ML: molecular layer, GL: granular layer, WM: white matter. Scale bar: 100 µm. (**c**) Rat cerebellum P30 sagittal section, the in utero grafted eGFP expressing hiPSdNP cells are located in the molecular layer, some of the cells were immunopositive for ATOH1 an early marker of cerebellar differentiation, their human nature was confirmed by positivity to hNu antigen. Abbreviations as in 1b. Scale bar: 30 µm. (**d**) Rat cerebellum P30 sagittal section, the image on the right is an enlargement of the left one and the asterisks mark the same position in the two pictures. hiPSdNP cells migrating away from the initial transplant site show more differentiated morphologies, some of them are immmunopositive for β-III tubulin but none has NeuN positive nucleus. Arrows indicate neurites positive both for β-III tubulin and eGFP. Abbreviation as in 1b and V: fourth ventricle. Scale bar for image on the left: 15 µm, for the enlargement on the right: 5 µm. (**e**) Another example of a successful transplant in P30 rat. Abbreviations as in 1b, arrow as in 1d. Scale bar: 15 µm. (**f**) Mouse cerebellum P7. There is a complete overlapping between EGFP and hNu positive cells indicating that all cells derive from the transplant, this is true also for cells that started to migrate away from the transplant, abbreviation as in 1b. Scale bar: 35 µm. (**g**) Mouse cerebellum P7. Complete overlapping between eGFP and hNu positivity is also present in bipolar cells that are scattered in the host cerebellum, abbreviation as in 1b. Scale bar: 25 µm. (**h**) Mouse cerebellum P7. Two cells derived from hiPSdNP with bipolar morphology, they appear SOX2 negative even if the field is rich in SOX2 positive host cells, arrowhead mark the position of the nuclei of the transplant derived cells that, when stained with Dapi, show a different degree of chromatin condensation compared to the host surrounding cells. Scale bar: 10 µm. (**i**) Mouse cerebellum P30, transplant P3. Graft derived cells are both clustered and dispersed into the host tissue. Morphological differentiation is more advanced in cells migrating away from the graft. Scale bars: 10 µm (left), 15 µm (right). (**j**) Mouse cerebellum P347. Starting from about one month post natal, only very few cells are eGFP positive, morphologically they differ from both glial and neuronal cells, being devoid of extensions, some of them appear binucleated (arrowhead), other have a chromatin condensation pattern that is undistinguishable from that of the host (arrow). Scale bar: 5 µm.

In order to estimate the rate of short term successful transplantation and rule-out any inhibitory effect of maternal immune response on transplant survival^26,27^, we analyzed the embryos of one pregnant mother per species before delivery. hiPSdNP cells were found scattered through the ventricles, associated with the choroid plexus and in the developing cerebellum and brainstem in 5 out of 8 mouse embryos (Fig. 1a). Of 63 mice and 25 rats born after receiving hiPSdNP cells into the cerebellum and fourth ventricles in utero, 14 mice and 3 rats were sacrificed during the first postnatal month, in this group: 6 out of 14 mice and 2 out of 3 rats showed a graft (Fig. 1b–h). None of the 49 mice and 22 rats sacrificed after one month of postnatal age showed sign of vital transplanted human cells (Table 1), differences in graft survival in the different temporal slots are statistically significant. Regardless from transplant modality, occasionally we found rare eGFP positive cells in the molecular layer of mice with very long transplantation survival. However, these cells were apparently devoid of glial processes or neurites; some were binucleated, and others had a single nucleus with a chromatin structure typical of mouse cells^28^ (Fig. 1i). Furthermore when tested immunohistochemically the cells were hNu negative further indicating that they were not human.

**Table 1:** Number of in utero transplanted animals with grafts containing healthy cells over all animals sacrificed during the post transplant time interval (days) indicated in the first column.

Days post natal	No. of surviving grafts/grafted mice	No. of independent mother mice	No. of surviving grafts/grafted rat	No. of independent mother rats
0–34	6/14	4	2/3	3
35–75	0/11	3	0/6	2
76–365	0/13	4	0/1	1
366–577	0/25	5	0/15	2

For each time interval the third and fifth columns indicate the number of independent pregnant female mice or female rats mothers to the sacrificed transplanted animals of each species. Siblings coming from the same mother were sacrificed at different time points. Differences between graft survival according to animal age groups were highly statistically significant (mice: χ^2^, 23.211; degrees of freedom (DF) 3, *p* < 0.0001; rats χ^2^, 15.942; DF 3; *p* < 0.0012).

### Survival of hiPSdNP transplanted into neonatal mouse is also limited

According to Billingham et al. (1958) tissues xeno-transplanted into early post-natal mice (before P6), like tissues xeno-transplanted into fetuses, have a good chance of inducing immuno-tolerance and survive into adulthood. The cerebellum of neonatal mice has the advantage that, despite still developing, can accommodate a cell inoculum without significant leakage. We stereotactically engrafted the same hiPSdNP we used for in utero transplantation into the cerebellar cortex and brainstem of 7 mouse pups (P0–P3). Again, we found viable transplanted human cells (2/3 mice) only up to the first month of life (Fig. 1j). At longer survival times (up to P56) we could not detect human cells in the host cerebellum or brainstem (4 mice).

### hiPSdNP grafted into the adult cerebellum of immunocompromised mice show long term survival

**Figure 2:**
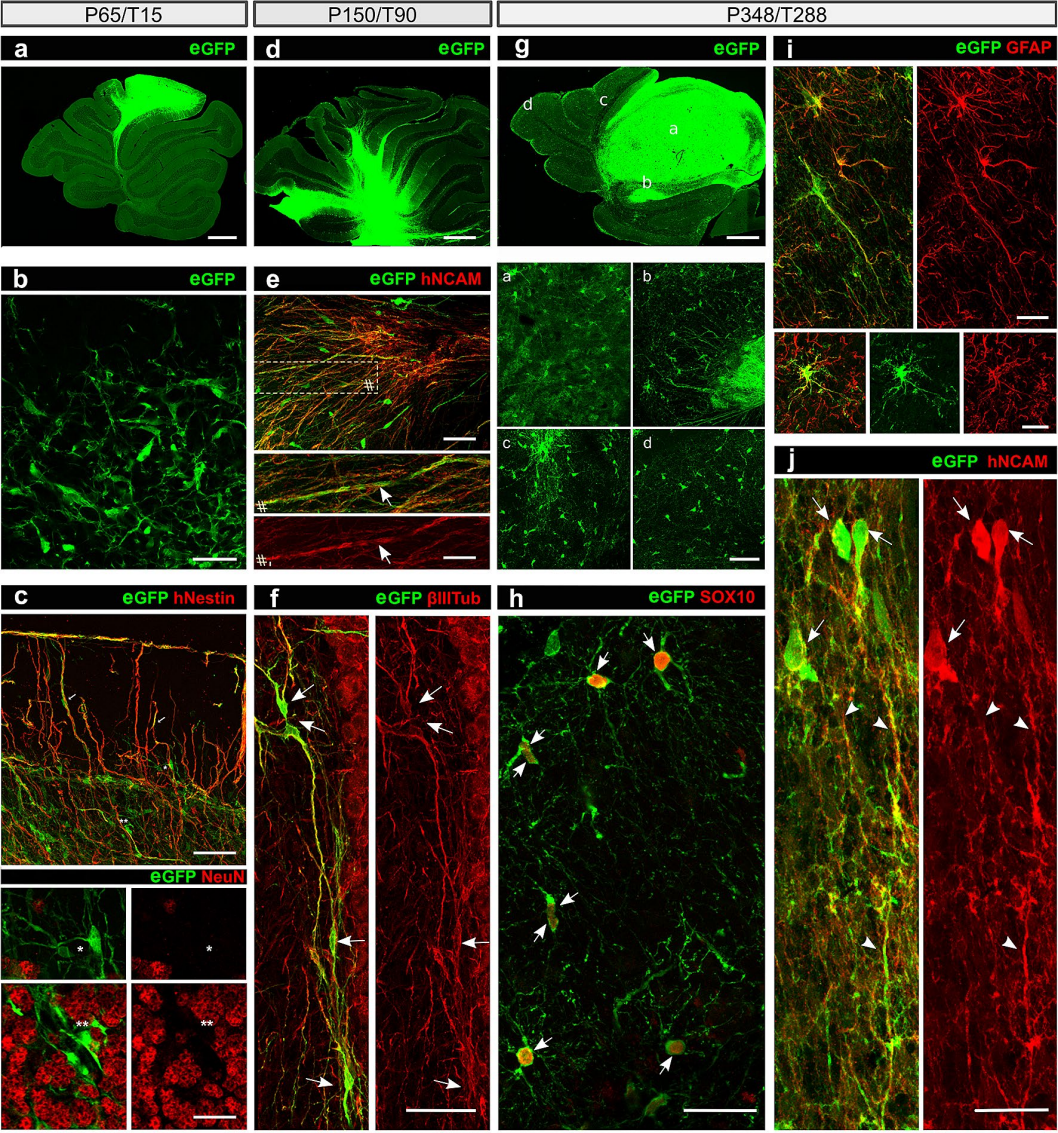
Representative photomicrographs of hiPSdNP transplanted into the cerebellum of adult NOD-SCID mice, time epochs are organized in columns. (**a**, **d** and **g**) Cerebellum sagittal sections. At increasing survival times the portion of cerebellum containing eGFP positive cells derived from the transplant increases due to cell dispersal and proliferation of neural precursors and glial cells. Scale bars: 100 µm. (**b**) Mouse cerebellum, at short survival times (15 days after the transplant) the majority of the engrafted cells maintain an undifferentiated morphology. Some cells bear distinct processes. Scale bar: 20 µm. (**c**) Mouse cerebellum survival as in B, sagittal section stained with anti hNestin (upper image) and anti NeuN (lower images) antibodies. Arrows mark examples of neurites double-labeled by antibody against eGFP and hNestin, asterisks indicate corresponding positions in all pictures, eGFP positive hiPSdNP derived cells are NeuN negative, while many of the surrounding host cells are NeuN immunopositive. Scale bars: 20 µm (upper), 10 µm (lower). (**e**) Mouse cerebellum 3 months after the transplant neuronal differentiation is ongoing with many process bearing cells immunopositive for hNCAM. The boxed area is enlarged at the bottom in order to show the close correspondence of eGFP and hNCAM positivity in neurites. Arrows indicate processes double positive for eGFP and hNCAM. Scale bars: 10 µm (upper), 5 µm (lower). (**f**) Mouse cerebellum 3 months after the transplant many neuronal cells are immunopositive for ß-III tubulin (arrows). Scale bar: 15 µm. (**g**) Mouse cerebellum 288 days after the transplant, higher magnification pictures of the areas labeled by the letters are provided below the low magnification image, showing the different morphologies and migration pattern of the cells derived from the transplanted hiPSdNP. Scale bars: 100 µm (upper image), 30 µm (lower four images). (**h**) Same survival as in G, many transplant derived cells differentiated into SOX10 positive cells (arrows). SOX10 is considered a marker of immature oligodendrocytes and myelinating oligodendrocytes^53^ Scale bar: 15 µm. (**i**) Same survival as in G, examples of eGFP positive hiPSdNP derived cells immunopositive for GFAP showing an astrocytic morphology. Scale bars: 10 µm. (**j**) Same survival as in G eGFP positive hiPSdNP derived neurons (arrows) positive for hNCAM with extensive processes (arrowheads) are also present at these survival times. Scale bar: 10 µm.

In order to test whether hiPSdNP were intrinsically unable of long term survival after xeno-transplantation into mice brain we transplanted the same cells into immunodeficient mice. We stereotactically transplanted hiPSdNP into the cerebellar vermis of 4 adult NOD-SCID mice (P60). All 4 xenotransplants survived; the cells were clustered together at short survival times, but progressively migrated out of the graft core to colonize the host tissue (Fig. 2a,d,g). Meanwhile, they differentiated into neurons and glia. Of note, and in line with the well-established capability of human glial progenitors to outcompete rodent cells^29,30^, we found that human glial cells had migrated throughout the whole cerebellum and part of the brainstem at the longest survival time (228 days after grafting) (Fig. 2g).

### In all transplant paradigms hiPSdNP increased expression of differentiation markers with time

Differentiation along the glial and neuronal lineages of the transplanted cells was progressive being maximal at the longest survival time. At the end of the first month post-transplant very few of the engrafted cells (Fig. 1b) were still immuno-positive for ATOH1 an early marker of cerebellar granule lineage that is lost after differentiation^31^, and they had lost the stem cell marker SOX2 (Fig. 1h). Transplant of hiPSdNP that in NOD-SCID mice survived beyond one month from grafting confirmed the trend to express more differentiated markers with increasing time from the transplant. Most transplanted cells (Fig. 2a,b) were still hNestin positive and NeuN negative (Fig. 2c) within one month from the transplant, while at later times they displayed either more differentiated neuronal markers like hNCAM and β-III tubulin (Fig. 2e,f,g,j) or glial markers typical of astrocytes and oligodendrocytes (GFAP and SOX10) (Fig. 2h,i).

### hiPSdNP and DhiPSdNP are both immunogenic in vivo

**Figure 3:**
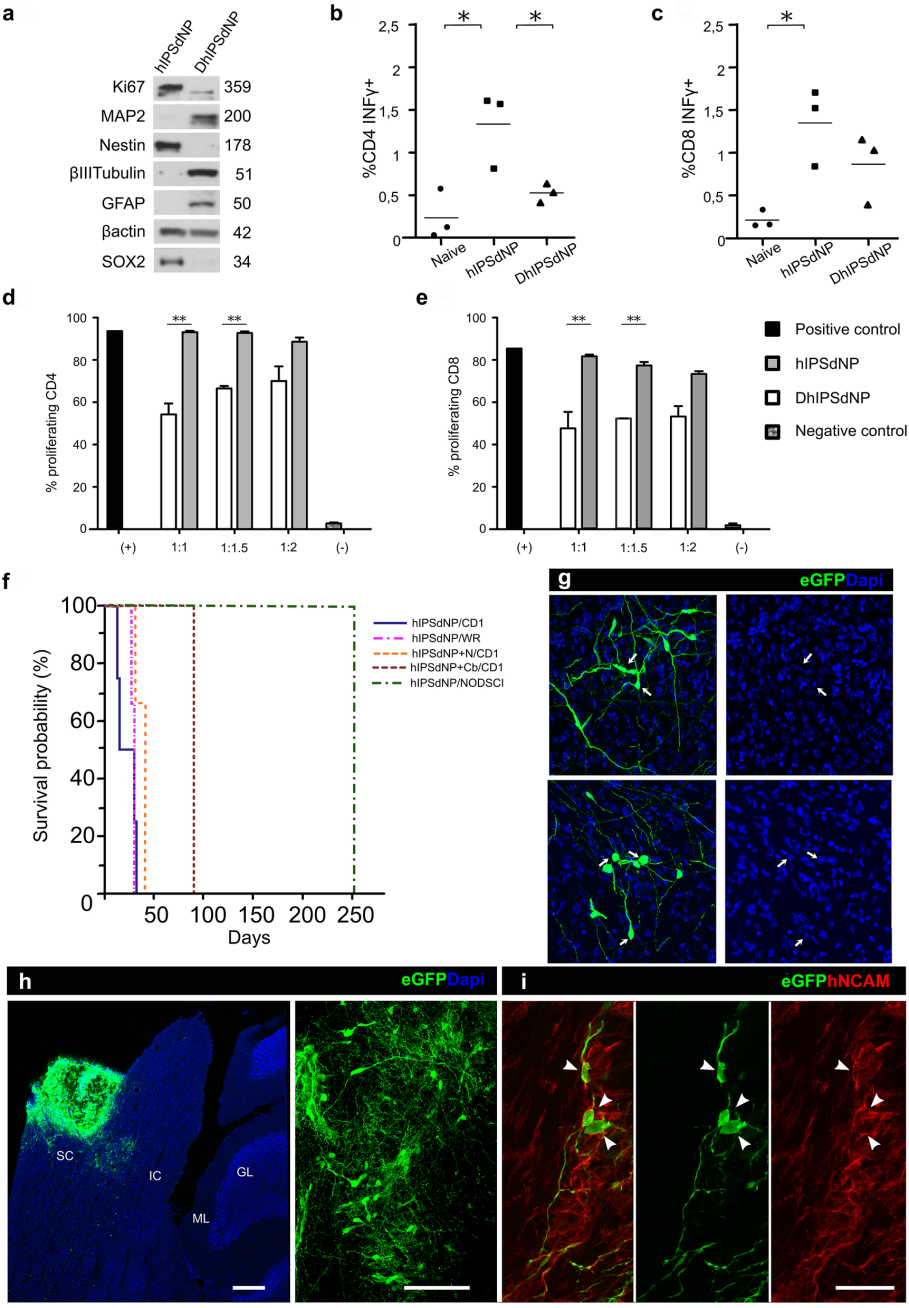
(**a**) Western blot analysis. After 55 days of in vitro differentiation treatment of eGFP expressing hiPSdNP the levels of antigens related to cellular proliferation and neural and/or glial differentiation change. The proliferation related Ki67 antigen decreases sharply in DhiPSdNP compared to hiPSdNP. Similarly, in DhiPSdNP Nestin and SOX2 both labeling neuro-glial precursors decrease, while MAP2 and β-III-tubulin that are present in postmitotic neurons, and GFAP, that is mainly present in astrocytes, increase. A housekeeping protein like β-Actin, remains the same. Numbers indicate the apparent molecular weight in kilodaltons. An uncropped image of the westerns used to assemble Fig. 3a is provided in Fig. S1. (**b**) Splenocytes from CD1 mice immunized with hiPSdNP or DhiPSdNP, or not immunized (Naive), were stimulated with PMA/ionomycin, in the presence of brefeldin A, and tested for IFNγ production by intracellular staining and flow cytometry. The histograms report the quantification of IFNγ production by CD4^+^ T lymphocytes; bars: average. Values are subtracted of background, i.e. spontaneous IFNγ release by unstimulated CD4^+^ or CD8^+^ T cells. N = 3 mice per group. One-way ANOVA followed by Tukey’s test: *p < 0.05. (**c**) Same as in B but the histogram shows the quantification of IFNγ production by CD8^+^ T lymphocytes. (**d**) hiPSdNP and DhiPSdNP were tested in vitro for suppressive activity against T cells. Responder CFSE-labeled splenocytes were stimulated with anti CD3 and anti CD28 antibodies, and left alone (+) or in the presence of hiPSdNP/DhiPSdNP, and tested after 5 days by flow cytometry. hiPSdNP/DhiPSdNP: responder ratio 1:1, 1:1.5 or 1:2 as indicated. Histogram reports percentage of proliferation for CD4^+^ T lymphocytes. Negative (−) controls were unstimulated splenocytes. The experiment was repeated three times. One-way ANOVA followed by Tukey’s test: **p < 0.005. (**e**) Same as in D but we measured proliferation of CD8^+^ T lymphocytes. (**f**) Kaplan–Meier analysis of survival of hiPSdNP transplanted in immunocompetent CD1 mice (CD1) and Wistar rats (WR) or immunodeficient mice (NOD-SCID) alone or as a mixture with unlabeled DhiPSdNP (hiPSdNP + N) or with adult rat cerebellar cells (hiPSdNP + Cb). (**g**) Mouse colliculum P45. Mice were transplanted at P3 with a mixture of EGFP labelled hiPSdNP and unlabelled DhiPSdNP. Dapi staining shows that the EGFP positive cells are mono-nucleated and their chromatin condensation pattern is very different (arrow) from that of the host cells (arrows). Scale bar: 10 µm. (**h**) Mouse cerebellum and brainstem 90 after transplant. The animal was transplanted in utero with a mixture of eGFP positive hiPSdNP and a suspension of cells derived by mechanical dissociation of adult rat cerebellum. Sagittal section eGFP and Dapi counterstained. The main cluster of transplanted cells is localized in the superior colliculus (SC) but eGFP positive cells are present also in the inferior colliculus (IC) and the brainstem. GL: granular layer, ML: molecular layer. Scale bar: 100 µm. On the right an enlargement of the area of the transplant. eGFP positive cells show different degrees of morphological differentiation and some display glial and neuronal morphologies. Scale bar: 25 µm. (**i**) Same as in h. Arrows indicate eGFP cells showing hNCAM immunopositivity. Scale bar: 15 µm.

To test hiPSdNP immunogenicity in vivo 9 naïve adult CD1 Swiss mice were immunized either with proliferating hiPSdNP or with the same cells submitted to an in vitro differentiation protocol that makes them post-mitotic (DhiPSdNP)^32^. After completing the in vitro differentiation treatment total protein extracts of DhiPSdNP contained higher levels of both neuronal maturation markers like β-III tubulin and MAP2 and SCG10 and glial markers as GFAP (Fig. 3a). Before i.p. injection, the cells were rendered apoptotic by treatment with camptotecin. Mice were killed one week later and tested for the activation of CD4 and CD8 T lymphocytes collected from the spleen by measuring their *ex-vivo* IFNγ production. Immunization with both cell types induced IFNγ production by T lymphocytes. This was higher in mice immunized with hiPSdNP (Fig. 3b,c). We also tested in vitro to ask if hiPSdNP or DhiPSdNP showed immunosuppressive activities on T lymphocytes. Only hiPSdNP but not DhiPSdNP were able to partially suppress the proliferation of murine CD4 and CD8 T cells (Fig. 3d,e).

### Co-grafting hiPSdNP and DhiPSdNP or a cellular extract of adult rat cerebellum extended survival of the xeno-grafted cells

Since hiPSdNP were unable to survive longer than a month even if transplanted before maturation of the host immune system in immune competent animals while they survived much longer in NOD-SCID mice, we hypothesized that the immune rejection of the transplanted hiPSdNP could be due to the expression of antigens that where not expressed in the transplant before the complete maturation of the host immune system. This was a consequence of the slower differentiation pace of the human cells that after the complete maturation of the host immune system, started to display new antigens linked to their progressive differentiation. Human antigens that were not expressed by the xeno-grafted cells during the maturation of the host immune system but started to be expressed later, were recognized by the host immune system as not-self triggering the immune-rejection of the xeno-grafted cells. To test this hypothesis, we co-xeno-grafted in the newborn (P3) cerebellum eGFP labeled hiPSdNP mixed with an equal amount of eGFP negative DhiPSdNP previously submitted to the same in vitro differentiation protocol used to generate DhiPSdNP for immunogenic testing^32^. In vitro differentiated DhiPSdNP became post-mitotic and expressed mature neural and glial antigens (Fig. 3a) although they did not develop into Purkinje cells or, Bergman Glia typical of the cerebellar cortex. The two animals transplanted with this mixture of cells developed transplants that survived up to P45 (Fig. 3f,g) showing a 43% increase in survival compared to the same cells transplanted alone.

We also co-xeno-grafted in utero eGFP expressing hiPSdNP mixed with a fresh cellular suspension obtained through mechanical dissociation of mature cerebella of adult Wistar rats in 24 fetuses carried by two pregnant mice. We used rat because we were not allowed to use human adult cerebellar tissue and adult rat is used as a substitute for human cerebellar tissue in clinical testing for the presence of auto-antibodies against human cerebellar antigens^33–35^. Most of the cells in the rat cerebellar extract (over 99%) had membrane damage as shown by positivity to the trypan-blue exclusion test before mixing. They were not intended to survive the transplant but only to provide fresh mature cerebellar antigens in the same context where the eGFP labelled hiPSdNP were grafted. Under these experimental conditions one xenograft over five mice sacrificed by three months survived up to P90 (Fig. 3h,i), longer than any xenografts of pure hiPSdNP. Nevertheless, survival was limited compared to immuno-deficient mice since we did not find any graft at survival times longer than three months in 19 siblings that received a similar graft before birth (Fig. 3f).

## Discussion

The results of our experiments indicate immune-rejection of hiPSdNP xeno-transplanted in utero or in early post-natal immunocompetent mice and rats about one month after transplantation. We sometimes found rare eGFP containing cells after the first month. However these cells were hNu negative, indicating that they were host cells. Both cell fusion events^21–23^ and transfer of eGFP to host cells^36^ have been previously described and could account for the presence in immune-competent animals of hNu negative eGFP containing cells at longer survival times. We observed indefinite survival of the same cells when transplanted into the cerebellum of immunocompromised mice. This suggest that the hiPSdNP were able to survive, migrate and differentiate after transplantation into the brain of mice when immune-rejection was inhibited. Moreover, lack of immune-attack to the hiPSdNP in utero followed by transplant survival up to P30 rule out any major effect of maternal immune response on xeno-transplant survival^26,27^. Mice neural precursors xenografted in the brain of adult immunocompetent rodents survive less than six weeks to the immune rejection response mounted by the host^37^. In contrast, xeno-grafting the same neural precursors into the developing cerebellum of rats and vice versa, results in viable transplants that integrate and differentiate into the host CNS without immunosuppressive therapy^19,38^. After in utero xeno-transplantion, engrafted mice cells and their descendants survive for the entire life of the host rat that tolerates the glial and neuronal antigens present in the cells derived from the donor species^19^. On the contrary, the in utero xeno-transplantation of hESC derived neural precursors into the developing brain of immuno-competent mice resulted in loss of all transplanted cells at the end of the first post natal month despite initial integration and migration of the transplanted cells into the host brain^25^. When undifferentiated hESC instead of hESC derived neural precursors were transplanted in utero into the developing mouse brain large intraventricular aggregates of undifferentiated cells together with more differentiated cells were still present months after the transplant^24^. However, the more differentiated cells dispersed in the brain could have migrated into the brain from the aggregates of less differentiated cells present in the ventricles long after the transplant^24^ making difficult to estimate their true post-transplantation survival.

These apparently contradictory results of xenografting in utero NP from different species into the developing brains of rats and mice may be reconciled by considering that NP are immature at the time of the transplant and start to differentiate in the host brain, following the developmental timing proper to the species of origin of the cells^3,19,39^. Maturation of hiPSdNP in vivo is approximately equal to nine months when transplanted into the cortex of immunocompromised mice^5^, while the same cells derived from rodents mature and are fully functional four to six weeks after transplantation^40,41^. If we consider that the temporal window for induction of immuno-tolerance to xenografted cells in mice and rats is limited to the fetal life and early postnatal period^10,13^ it follows that the long time required for differentiation of hiPSdNP after transplantation may limit tolerance induction only to those antigens expressed by the xeno-grafted cells during the temporal window for immuno-tolerance induction. In contrast, antigens that the cells derived from the hiPSdNP will only start to express later, will be new for the host and thus able to elicit immune response causing the immune-rejection of the graft. Mitochondrial neoepitopes that may originate de novo in hiPSC *in vitro*^42^ in our experimental settings are unlikely to have influenced survival of hiPSdNP after in utero xeno-transplantation, since, if present, they should have been already present in mitochondria of the hiPSdNP at the time of transplantation. Thus, being expressed before complete maturation of the immune system they would have induced immuno-tolerance^43^.

In agreement with our hypothesis that human antigens that started to be expressed after the maturation of the immune system were responsible for the immune rejection of the transplanted hiPSdNP after their initial taking, is that the same hiPSdNP that we used for in utero grafting, differentiated and survived indefinitely in the cerebellum of immunodeficient mice. These cells and their derivatives progressively lost early antigens labeling neuronal or glial progenitors like nestin, and ATOH1 and around P30 started to express more differentiated antigens like ß-III tubulin, NCAM, GFAP, and SOX10. In immunocompromised mice the maturation process together with extensive migration of the cells homing in portions of the cerebellum and brainstem away from the original sites of transplant, progressed in the following months, while all grafted cells disappeared in immune-competent animals around the end of the first month. Immune rejection by activation of cellular immunity after allogenic transplantation of iPSC-derived neurons is well documented in rodents and primates^44^.

Our results indicate a small but significant extension of transplant survival by co-grafting hiPSdNP and an aliquot of the same cells after in vitro differentiation (DhiPSdNP) in to more mature glial and neural cells. This observation supports the idea that immune tolerance to hiPSdNP is normally induced by transplanting the cell in utero but the induced tolerance does not protect the engrafted cells from the immune response to antigens that started to be expressed much later according to the human developmental timing. In line with this hypothesis we were able to further extend the survival of the xenografted-cells by transplanting in mice in utero hiPSdNP mixed with an extract containing cells and protein from normal adult rat cerebellum.

We used rat cerebellum since human cerebellar tissue is not easily available and rat tissue is already used as a substitute for human cerebellar tissue in clinical testing for the presence of auto-antibodies against cerebellar cells^33–35^. Our results suggest that more differentiated antigens present in the adult rat cerebellum are sufficiently homologous to human neural cells to enhance immuno-tolerance of the host mice, in some cases delaying the immune-rejection of the transplant triplicating maximal survival of the cells. The partial protective effect of the xenogenic adult cerebellar suspension co-injected with hiPSdNP may have been enhanced by the simultaneous exposure of the developing immune system of the host to mature intracellular and membrane exposed antigens. That, may have enhanced both peripheral and central mechanism of negative selection of clones targeting cerebellar antigens that, at least in humans, are the most common targets for autoimmune attacks^45^. This suggests in turn that even proteins not related to the human HLA complex, may also be crucial for long term immune-tolerance of the graft.

Our results also indicate that, at least in vitro, hiPSdNP but not their more differentiated derivatives, were able to partially suppress the proliferation of murine CD4 and CD8 T cells. An immunosuppressive activity mediated by TGFß has also been described for NP derived from primate IPSC cells^44^. The direct immunosuppressive activity of hiPSdNP may have complemented the immuno-tolerance induced by xeno-transplantation of the cells before complete maturation of the host immune system in the initial xeno-graft survival. However, the intrinsic hiPSdNP immunosuppressive activity alone cannot promote definitive xenograft survival when the hiPSdNP are xeno-transplanted into the CNS of a mature host^7^.

There is a growing interest for clinical applications of in vitro differentiated hiPSdNP cells to neurotransplantation paradigms and the importance of controlling the response of the host immune system is widely recognized as essential to allow significant transplant survival^46,47^. Our data indicate that hiPSdNP transplanted in a xenogenic contest may initially survive but that with progressive expression of more differentiated neural and glial antigens the induced partial immuno-tolerance is overcome and the host immune system attacks the xenogeneic cells rejecting the transplant. These findings confirm that developmental timing is a cell autonomous process strictly dependent on the species of origin. They also stress the importance of considering the evolving nature of the transplant as an important factor to predict the host immune response. This factor should also be taken into account in planning the duration of immunosuppressive therapy for experimental and clinical applications of neural transplantation.

## Methods

### Cells used for transplantations

hiPSdNP cells were previously described and indicated as healthy donor-derived Neural Precursor Cells (WT1-NPCs)^32^. Briefly they were obtained from hiPS clones generated from integration-free reprogrammed fibroblasts (Coriell Institute Biobank, Camden, New Jersey, USA). After hiPS colony isolation and expansion, a selected hiPS clone was exposed to PSC Neural Induction Medium (Thermo Fisher Scientific) for seven days. The induced cells were subsequently dissociated with Accutase (Carlo Erba Reagents) and seeded on Geltrex pre-coated flasks in Neural Expansion Medium (50% Neurobasal Medium, 50% Advanced DMEM F-12 and 2% Neural Induction Supplement, all from Thermo Fisher Scientific) to derive hiPSdNP. eGFP positive stable subclones of hiPSdNP were obtained by infection with lentiviral vector obtained by cloning the eGFP cDNA in the pLenti-GFP lentiviral vector^48^. After infections clones of hiPSdNP expressing eGFP were isolated and used as source of cells for transplantation. Cells used for transplantation were dissociated with Accutase, harvested by centrifugation for 7 min at 0.8G counted, resuspended in 10 ml of fresh medium and maintained at 4 °C till transplantation.

### In vitro differentiation of hiPSdNP into DhiPSdNP

hiPSdNP cells were differentiated in vitro according to the protocol of Yan et al^49^. During this in vitro differentiation protocol hiPSdNP stop dividing and become post-mitotic^32^. hiPSdNP were seeded at a 5 × 10^4^ cells/cm^2^ on twofold concentrated Geltrex (Thermo Fisher Scientific) pre-coated plates and kept in Neural Differentiation Medium (Neurobasal medium, 2% B27 supplement serum free, 2 mM GlutaMAX supplement, from Thermo Fisher Scientific, 10 ng/μl brain derived neurotrophic factor and 10 ng/μl glial cell-derived neurotrophic factor, both from Immunotools, 200 μM L-ascorbic Acid from Merck, 0.1 mM non essential amino acids, 100 units/ml penicillin and 100 μg/ml streptomycin from Lonza) up to 55 days in vitro. Their differentiation of neural and glial pathways was followed by western blotting.

### Adult cerebellar extracts

Two adult Wistar rats were deeply anesthetised with a solution of ketamine (100 mg/kg Ketavet, Gellini) and xylazine (33 mg/kg Rompun, Bayer) and decapitated. The cerebellum was dissected off the cranium, immersed in cold (4 °C) PBS, freed from the meninges and the cerebellar cortex quickly minced in blocks of approximately 1 mm in diameter, further dissection was carried out by aspirating the fragments through a series of pipette tips of decreasing diameter and left to decant in ice for at least 10 min. The supernatant was aspirated and used for co-transplantation after counting the number of resuspended cells. Trypan blue exclusion test carried put on the suspension indicated that the large majority (over 98%) of the cells were unable to exclude the dye.

### Surgical procedures and animal strains

All procedures were performed in accordance with the European Communities Council Directive (and 86/609/EEC and 2010/63/EU), the National Institutes of Health guidelines, and the Italian Law for Care and Use of Experimental Animals (d. lgs. 26/2014). They were approved by the Italian Ministry of Health and the Bioethical Committee of the University of Turin (Project “Invecchiamento, degenerazione e crescita tumorale nel sistema nervoso centrale.” 741/2016 PR to AB). The study was conducted according to the Arrive guidelines.

In utero transplantations in immunocompetent animals were performed in 5 pregnant Wistar rats at embryonic day 16 (E16) and 2 pregnant at E18 (RccHan:WIST), and in 12 CD-1 pregnant E14 females mice (Hsd:ICR; Envigo). The day of vaginal plug detection was defined as E0, and the day of birth was considered as P0^50^. Postnatal transplantations of hiPSdNP into immunocompetent-mice were performed before P4 in 7 CD-1 mice (Hsd:ICR; Envigo).

Transplantations into immune-deficient animals were performed in 4, 8–12 week old NOD-SCID mice (NOD.CB17-Prkdcscid/NCrHsd; Envigo). All animals were maintained on a 12-h day/night cycle with adequate food and water.

Transplantation in adult immunocompetent mice to study the in vivo immunogenicity of hiPSdNP and DhiPSdNP were performed in 11 CD-1: 9 transplanted and 2 controls, all were 8 weeks old.

All surgical procedures were performed under deep general anesthesia. Pregnant and adult animals were anesthetized by 1–2.5% of isoflurane in O_2_ in a mixture of O_2_/N_2_O (30:70). Newborn CD1 pups were anesthetized by hypothermia on melting ice.

### In utero transplantation

We performed in utero transplants into rat and mouse fetuses as previously described^51^. Briefly, after anesthesia we exposed the uterine horns of time pregnant rats or mice thorough a limited midline incision of the abdomen. After identification of the developing cerebellum and fourth ventricle by transillumination, we injected with a glass capillary 2 µl of a cell suspension containing 5 × 10^4^ hiPSdNP into the developing cerebellum or brainstem. After returning the uterus into the abdomen we sutured the abdominal wall with non-absorbable sutures.

### Postnatal transplantation

Mice were anesthetized and positioned in a stereotaxic apparatus (Stoelting). We exposed the posterior surface of the cerebellum by limited drilling of the occipital bone, and stereotactically injected in 2 min, 2 μl of the cell suspension (5 × 10^4^/µl) through a glass micropipette attached to a pneumatic pressure injection apparatus (Picospritzer II, General Valve Corporation), at the end of the injection the micropipette was removed, the wound sutured, and the animal was returned to its cage. Stereotactic coordinates for transplantation were for neonatal mice: AP (from bregma) − 4.5 mm; LAT (from midline) 1.2 mm, Depth (from cranium) − 3 mm; for adult AP (from bregma) − 7 mm; LAT (from midline) 2 mm, Depth (from cranium) − 5 mm.

### Tissue preparation

Tissues were prepared according to standard methods^38^. Briefly, after induction of anesthesia with a solution containing 100 mg/kg of ketamine (Ketavet, Gellini) and 33 mg/kg of xylazine (Rompun, Bayer). When deeply anesthetized the animals were perfused with a solution containing 4% paraformaldehyde (PFA) and 2% picric acid (AnalytiCals, Carlo Erba 409302) in 0.1 M sodium phosphate buffer (PB) pH 7.4. After dissection brains were post-fixed for 3 h, cryoprotected in 30% sucrose (S0389 Sigma) in 0.1 M PB pH 7.4, embedded at − 80 °C in Killik/OCT (Bio-Optica 05-9801), and serially cut on a cryostat, into 40 µm-thick sections.

### Immunofluorescence

Immunofluorescence was performed as described before^38,50^, briefly: sections were incubated for 48 h at 4 °C in 0.01 M PBS pH 7.4 containing 2% Triton X-100 (TBS), 1:100 normal donkey serum and the primary antibodies. Sections were incubated overnight with appropriate secondary antibodies, extensively washed in TBS and mounted in antifade mounting medium Mowiol (4–88 reagent, Calbiochem 475904). The following primary antibodies and dilutions were used: chicken anti-GFP, 1:000 (AvesLabs, GFP-1020); rabbit, polyclonal anti-GFAP 1:1.500 (Dako Z0334), rabbit anti-hNESTIN, 1:500 (Millipore, ABD69); mouse anti-hNCAM, 1:200 (Santa Cruz, sc-106); mouse anti hNu, 1:1000 (Millipore, MAB128); mouse anti-NeuN, 1:1000 (Chemicon, MAB377); goat anti-SOX10, 1:1000 (Santa Cruz, sc-17342); mouse anti-β-III tubulin (Tuj1), 1:500 (Sigma, T8660). The following secondary antibodies and dilutions were used: donkey anti-rabbit (Cy3 labeled, 1:800, Jackson ImmunoResearch, 711-165-152; donkey anti mouse (Cy3 labeled, 1:800, Jackson ImmunoResearch 715-165-151); donkey anti goat (Cy3 labeled, 1:800, Jackson ImmunoResearch 705-165-147); donkey anti chiken (AlexaFluor488 labeled, 1:400, Jackson ImmunoResearch 703-545-155).

### Histological analysis

After staining of the sections (40 µm-thick) with an anti eGFP antibody we examined all sections for eGFP-positive cells present in the cerebellum and brainstem of the transplanted animals with a Nikon Eclipse 801 microscope equipped with a Nikon TV lens C-0.6X digital camera. Approximately 170 sagittal sections for each adult rat and 105 for each adult mouse were examined to cover entirely the brainstem and cerebellum. Quantitative and phenotypic evaluations were made on images acquired with a Leica TCS SP5 confocal microscope. Fiji (http://fiji.sc/Image_Stitching), Inkscape (http://inkscape.org), and Photoshop CS6 (Adobe Inc. https://www.adobe.com) were used to assemble all figures.

### Immunization protocol

hiPSdNP and DhiPSdNP were induced to apoptosis by treatment with a solution containing Camptotecin (Sigma) 3 μM for 12 h before injection. Then, 2 × 10^6^ cells were injected i.d. into the right flank of naïve Swiss mice. Seven days later mice were sacrificed and their splenocytes were analyzed by flow cytometry for IFNγ production by CD4 and CD8 T cells. The following primary antibodies were used: anti mouse IFNγ APC clone XMG1.2, (eBiosciences cat no. 17-7311-82), anti mouse CD4 BV650 clone RM4, (Biolegend cat no.100545), anti mouse CD8a PE-Cy7 clone 53-6.7 (eBiosciences cat no. 25-0081-82).

### In vitro suppression assay

10^5^ naïve murine splenocytes were labeled with a solution containing 1.5 μM Carboxyfluorescein succinimidyl ester (CFSE) (eBiosciences) and cultured alone or with hiPSdNP or DhiPSdNP at different ratios, in the presence of 2 mg/ml of anti-CD3 and 1 mg/ml of anti-CD28 (eBiosciences) to activate lymphocytes. Proliferation of CD4 and CD8 T cells was assessed 5 days later, evaluating CFSE dilution by flow cytometry.

### Flow cytometry

Flow cytometry was performed according to standard methodology^52^. Briefly, spleen cell suspensions were obtained from mechanical disaggregation of murine spleens. Cells were incubated 10 min with FcR blocker and labeled for 15 min at 4 °C with fluorochrome-conjugated monoclonal antibodies (all from eBiosciences or BioLegend). For intracellular detection of IFNγ, splenocytes were stimulated 4 h with phorbol 12-myristate 13-acetate (PMA; 120 ng/ml, Sigma) and ionomycin (1 μg/ml, Sigma), adding brefeldin A (10 μg/ml, Sigma) in the last 3 h, as described^52^. Cells were stained for surface markers, fixed with 2% PFA, permeabilized with saponin (0.5% in PBS) and incubated with anti IFNγ antibody. Samples were acquired with BD LSRII Fortessa and analyzed with the FlowJo software (Version 10.6 Ashland, OR, USA, https://www.flowjo.com/solutions/flowjo/downloads). The following primary antibodies that were not mentioned before were also used: anti mouse CD4 PE clone GK1.5, eBiosciences cat no. 12-0041-82, anti mouse CD8a BV605 clone 53-6.7 (Biolegend cat no. 100743).

### Protein extraction and western blot analysis

Total protein extracts were obtained from hiPSdNP and DhiPSdNP after detaching the cells with accutase, cells were then washed and lysed in 20–100 μl of lysis buffer (0.125 M Tris-HClpH 6.8, 5% SDS) containing proteases and phosphatases inhibitors. For western blot analysis 30 μg of total protein extracts were loaded on Novex NuPAGE precast gels, electrophoresed and blotted onPVDF membranes (Merck). After blocking in 4% non-fat dry milk, membranes were incubated with the appropriate primary antibodies overnight at 16 °C using the X-BLOT P100 System (Isenet, Milano, Italy), then for 1 h with the appropriate horseradish peroxidase-conjugated secondary antibodies (GE Healthcare). Proteins were detected by chemiluminescence and signals quantitated by densitometric analysis using the Image Quant software (version 5.2, Cytiva; https://cytivalifesciences.com). Total protein loading per lane was normalized with antibodies against β-Actin. The following primary antibodies that were not mentioned before were used for western blotting included: mouse monoclonal anti-Ki-67 1:1000 (Agilent Dako #M7240); mouse monoclonal anti-MAP2 Merck 1:5000 (Millipore MAB3418); mouse monoclonal anti-nestin 1:1000 (Merck Millipore MAB5326); mouse anti-βIII-Tubulin (TUJ1) 1:10,000 (Sigma-Aldrich T8578); mouse anti-GFAP 1:2000 (Merck Millipore AB5804); mouse monoclonal anti-β-Actin 1:20,000 (Sigma-Aldrich A1978); mouse monoclonal anti-SOX2 1:1000 (Cell Signaling Technology 3579).

### Statistical analysis

Transplant survival was calculated using the date the date of sacrifice of the host. Survival curves were obtained by Kaplan–Meier analysis, and significant differences in transplant survival were evaluated by the log-rank test by using. Considering that our goal was to compare xenograft survival beyond one month in immunodeficient versus immunocompetent mice, we estimated the sample size of immunodeficient animals (positive control) using Fisher exact test for proportions with commonly accepted values for significance (type one error, alpha) of 0.05 and power (type II error, beta) of 0.20 proportion of grafts surviving beyond one months in immunodeficient vs. immunocompetent mice of 100% and 0%. With those parameters the sample size results of 3 immunodeficient and 5 immunocompetent mice with a ratio of sample size for the two groups of 0.66. The animals we effectively employed were slightly higher (7 immunocompetent and 4 immunodeficient mice) since one mouse in the immunodeficient group and two in the immunocompetent one were sacrificed before one month. All calculations except those for immunogenicity and immunosuppressive assays were performed using MedCalc Statistical Software version 18.2.1 (MedCalc, Ostend, Belgium; http://www.medcalc.org; 2018). For immunogenicity and immunosuppressive assays statistical comparisons were performed with ANOVA followed by Tukey’s tests, using the GraphPad Prism software (version 8.1.0, GraphPad Software, La Jolla, CA, USA; https://www.graphpad.com). Differences were considered significant when *p* < 0.05 (*) or* p* < 0.005 (**).

### Ethics declarations

The animal protocols were approved by the Italian Ministry of Health and the Bioethical Committee of the University of Turin (Project “Invecchiamento, degenerazione e crescita tumorale nel sistema nervoso centrale.” 741/2016 PR to AB). The study was conducted according to the Arrive guidelines.

## Supplementary Information

Supplementary Figure.
