# Supplementary figures and images for: Immune-tolerance to human iPS-derived neural progenitors xenografted into the immature cerebellum is overridden by species-specific differences in differentiation timing
Source: Sci Rep. 2021 Jan 12;11:651. doi: 10.1038/s41598-020-79502-9 (PMC7803978; doi:10.1038/s41598-020-79502-9)

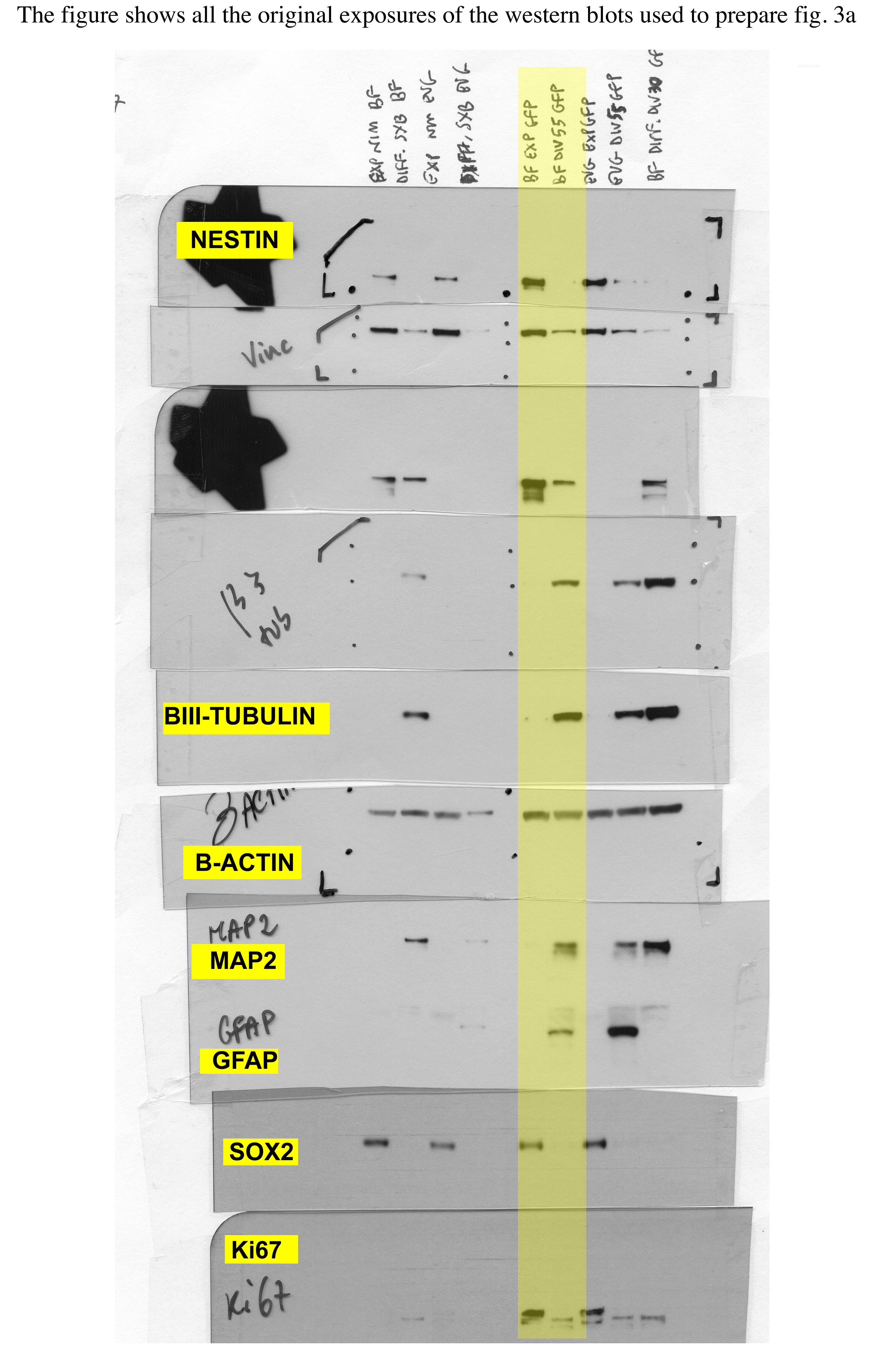

Supplement: Supplementary file 1 — Supplementary Figure. [file 41598_2020_79502_MOESM1_ESM.jpg]
